# Development of an algorithm using clinical tests to avoid post-operative residual neuromuscular block

**DOI:** 10.1186/s12871-017-0393-4

**Published:** 2017-08-04

**Authors:** Christoph Unterbuchner, Manfred Blobner, Friedrich Pühringer, Matthias Janda, Sebastian Bischoff, Berthold Bein, Annette Schmidt, Kurt Ulm, Viktor Pithamitsis, Heidrun Fink

**Affiliations:** 1 Klinik für Anaesthesiologie, Klinikum rechts der Isar, Technische Universität München, Munich, Ismaninger Str. 22, 81675 Munich, Germany; 2 Klinik für Anaesthesiologie, Universitätsklinikum Regensburg, Universität Regensburg, Franz-Josef-Strauss-Allee, 11 93051 Regensburg, Germany; 3 Klinik für Anaesthesiologie und operative Intensivmedizin, Klinikum am Steinenberg, Steinenbergstr. 31, 72764 Reutlingen, Germany; 4 Klinik und Poliklinik für Anästhesiologie und Intensivtherapie, Universität Rostock, Schillingallee 35, 18057 Rostock, Germany; 5 Klinik und Poliklinik für Anästhesiologie und operative Intensivmedizin, Universitätsklinikum Münster, Albert-Schweitzer-Campus 1, 48149 Münster, Germany; 6 Klinik für Anästhesiologie und Operative Intensivmedizin, Universitätsklinikum Schleswig-Holstein, Campus Kiel, Arnold-Heller-Str. 3, 24105 Kiel, Germany; 7 Klinik für Anaesthesiologie, Universitätsmedizin der Johannes Gutenberg-Universität Mainz, Langenbeckstr. 1, 55131 Mainz, Germany

**Keywords:** Postoperative residual curarization, electromyography, acceleromyography, algorithm, clinical muscle function tests

## Abstract

**Background:**

Quantitative neuromuscular monitoring is the gold standard to detect postoperative residual curarization (PORC). Many anesthesiologists, however, use insensitive, qualitative neuromuscular monitoring or unreliable, clinical tests. Goal of this multicentre, prospective, double-blinded, assessor controlled study was to develop an algorithm of muscle function tests to identify PORC.

**Methods:**

After extubation a blinded anesthetist performed eight clinical tests in 165 patients. Test results were correlated to calibrated electromyography train-of-four (TOF) ratio and to a postoperatively applied uncalibrated acceleromyography. A classification and regression tree (CART) was calculated developing the algorithm to identify PORC. This was validated against uncalibrated acceleromyography and tactile judgement of TOF fading in separate 100 patients.

**Results:**

After eliminating three tests with poor correlation, a model with four tests (*r* = 0.844) and uncalibrated acceleromyography (*r* = 0.873) were correlated to electromyographical TOF-values without losing quality of prediction. CART analysis showed that three consecutively performed tests (arm lift, head lift and swallowing or eye opening) can predict electromyographical TOF. Prediction coefficients reveal an advantage of the uncalibrated acceleromyography in terms of specificity to identify the EMG measured train-of-four ratio < 0.7 (100% vs. 42.9%) and <0.9 (89.7% vs. 34.5%) compared to the algorithm. However, due to the high sensitivity of the algorithm (100% vs. 94.4%), the risk to overlook an awake patient with a train-of-four ratio < 0.7 was minimal. Tactile judgement of TOF fading showed poorest sensitivity and specifity at train of four ratio < 0.9 (33.7%, 0%) and <0.7 (18.8%, 16.7%).

**Conclusions:**

Residual neuromuscular blockade can be detected by uncalibrated acceleromyography and if not available by a pathway of four clinical muscle function tests in awake patients. The algorithm has a discriminative power comparable to uncalibrated AMG within TOF-values >0.7 and <0.3.

**Trial registration:**

Clinical Trials.gov (principal investigator’s name: CU, and identifier: NCT03219138) on July 8, 2017.

## Background

Use of neuromuscular monitoring together with pharmacological reversal of neuromuscular blocking drugs in the operation room is able to reduce the incidence of residual paralysis in patients arriving in the postoperative care unit (PACU), especially when a quantitative monitoring device is used [1, 2]. Unfortunately, quantitative neuromuscular monitoring is neither available in many operating rooms nor regularly used worldwide [3–6]. Many anaesthesiology societies do not reinforce quantitative neuromuscular monitoring [7–9]. Therefore, in everyday practice, anaesthesiologists often prefer simple peripheral nerve stimulators (PNS) to assess fading qualitatively. However if there is no tactile fading in TOF and double burst stimulation a 50% risk of actual TOF ratio < 0.7 remains. Appeals to use quantitative techniques on a routine basis are not heard and lead to more resistance rather than compliance [10–12]. This dilemma will most likely not change fundamentally in the near future. Therefore, we will continue to see patients with residual neuromuscular block in the PACU.

Neuromuscular monitoring in awake patients has so far not been validated [13]. Rejection of quantitative neuromuscular monitoring in the PACU is, therefore, more understandable than its widespread intraoperative denial. In other words, it cannot be expected that anaesthetists, who do not rely on quantitative neuromuscular monitoring ***during*** anaesthesia, change their approach ***after*** anaesthesia. Nevertheless, studies investigating postoperative residual paralysis used neuromuscular monitoring in a postoperative setting [14–18]. Although these studies could demonstrate accordance between acceleromyography (AMG) and clinical signs of muscle weakness, there is no standard diagnostic tool for postoperative residual neuromuscular block [14–18].

In this multicentre, prospective, double-blinded, assessor controlled study we developed and validated an algorithm of clinical muscle function tests to identify residual paralysis in awake patients after anaesthesia. First, we tested a set of clinical muscle function tests with train-of-four ratio (TOFR) measured simultaneously by calibrated electromyography (EMG) at the adductor pollicis muscle. Second, the battery of tests was reduced to an algorithm, which becomes applicable in a clinical setting. Finally, an uncalibrated AMG, the algorithm, and qualitative tactile neuromuscular monitoring were validated in a separate set of awake patients by comparison with calibrated EMG.

## Methods

### Patients

**Fig. 1:**
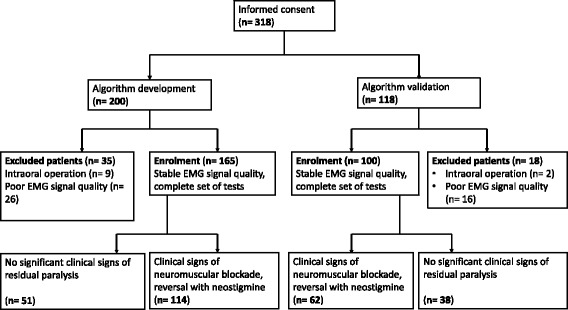
CONSORT Flow Diagram

After approval by the local ethics committees (main ethical committee, Technische Universität München, Germany; protocol N° 1783/07) of the six participating study centres and written informed consent, 318 patients (ASA 1–2) were enrolled in the study. The patients were scheduled for elective low risk surgical procedures (e.g. laparoscopic abdominal procedures; orthopedic and minor visceral surgery). Patients were excluded from the study if they currently participated in another study, if their body mass index was over 30, if age was under 18 or over 65 years, if they had a history of neuromuscular diseases or gastro-esophageal reflux disease. A set of 200 patients served as data pool to develop the algorithm of clinical muscle function tests to identify residual neuromuscular block. In another set of 118 patients, the developed tool was validated (Fig. 1, CONSORT flow diagram). Study was performed between 2008 and 2009.

### Procedure

After arrival to the operating room, standard monitoring was applied which included electrocardiography, pulse oximetry, automatic non-invasive blood-pressure measurement, oropharyngeal temperature and capnography. Patients were anaesthetized with remifentanil and propofol and ventilated with 100% oxygen using a facemask.

Neuromuscular function was monitored according to international consensus guidelines, using evoked EMG of the adductor pollicis muscle with a NMT module in a S/5 GE Datex Light monitor (GE Datex Medical Instrumentation, Inc., Tewksbury, MA, USA) by a non-blinded investigator [19]. The blinded anaesthesiologist was unable to see the data on the monitor. In brief, the forearm was immobilised and surface skin electrodes were placed over the ulnar nerve along the forearm. Following calibration, the ulnar nerve was stimulated with supramaximal train-of-four (TOF) stimulation at 20s intervals and the evoked electromyogram of the adductor pollicis muscle was recorded. The neuromuscular transmission and its suppression is described by parameters related to the TOF stimulation patterns, i.e., the response to the first stimulation related to the baseline values (T1/T0) and the TOFR.

Following 3 min of stabilization of the EMG recording, atracurium 0.5 mg/kg was intravenously injected and the trachea intubated. Anaesthesia was maintained with remifentanil 0.05–1.0 μg/kg/min and desflurane 0.9–1.1 MAC (age adapted) in 40–50% oxygen. Ventilation was controlled to maintain normocapnia (35–45 mmHg). During surgery, the (blinded) attending anaesthesiologist administered maintenance doses of atracurium 0.1 mg/kg according to clinical needs, i.e. without knowing the TOFR. Oropharyngeal temperature was kept ≥36 °C by forced air warming devices.

After end of surgery, the blinded anaesthesiologist stopped remifentanil infusion and desflurane inhalation. The patients’ trachea was extubated according to clinical judgement (sufficient alertness, cooperation, sufficient spontaneous ventilation), without knowledge of the quantitatively monitored EMG values.

Immediately after extubation the blinded anaesthesiologist tested the patient in the operating room. The postoperative evaluation of neuromuscular function consisted of eight clinical tests applied in a random order (Table 1). Thereafter, an uncalibrated AMG was started on the contralateral arm (TOF-Watch-Monitor; MIPM GmbH, Mammendorf, Germany). To avoid movement artefacts, the patient’s arm and the other four fingers were fixed to the arm rest. The response of the adductor pollicis muscle to ulnar nerve TOF stimulation (50 mA, 2 Hz, 200 ms) was measured. During the validation of the developed algorithm the anaesthesiologist had to judge tactile fading of the adductor pollicis before the acceleration transducer was applied to the distal phalanx of the thumb.

**Table 1:** Clinical tests to evaluate the neuromuscular function. The tests were performed after extubation in the awake, alert, and cooperative patient

Test	Evaluation	Scores
Open eyes	Time able to keep eyes open [s]	0–5
Diplopic image	Appearance of diplopic images [yes = 0; no = 1]	0–1
Stick out tongue	Time able to stick out tongue [s]	0–5
Spatula pressure	Subjective strength necessary to pull out the spatula against the patient’s occlusion efforts	0–3
Head lift	Time able to elevate the head from the pillow in supine position [s]	0–5
Arm lift	Time able to elevate the arm to 45° in supine position [s]	0–5
Press hand	Subjective strength of the patient pressing the investigator’s hand	0–3
Swallowing 20 ml of water	Impossible = 0, possible with choking = 1, possible, but with problems = 2, possible without any hindrance = 3	0–3

**Fig. 2:**
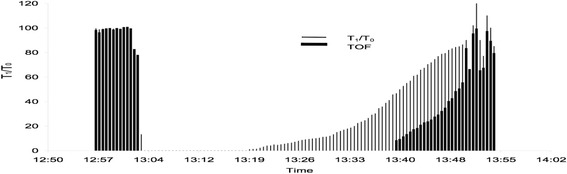
Example of an instable EMG signal during return of consciousness. After moving the arm the cable was disconnected. The patient did not accept connecting the EMG again or the AMG on the contralateral arm

Neuromuscular monitoring in awake patients is prone to artefacts. Therefore, a second blinded anaesthesiologist thoroughly reviewed the EMG files afterwards. In patients where either no data could be obtained or the TOFR dropped by more than 20% at least twice during the clinical muscle function tests, the respective data was excluded from analysis (Fig. 2).

If a patient had any clinical signs of neuromuscular dysfunction, reversal with neostigmine 40 μg/kg preceded by glycopyrrolate 7 μg/kg was administered. Due to safety issues, the clinical muscle function tests and the AMG measurements were repeated within 30 min. Then, patients were transferred to the PACU, where circulatory, cardiac, and respiratory function were monitored for at least 2 h before the patients were discharged from the PACU.

### Data management and statistical analyses

We compared tools (uncalibrated AMG, tactile evaluation following peripheral nerve stimulation and algorithm of muscle function tests) with a gold standard (calibrated EMG). Accordingly, we only analysed patients’ data whose EMG signal was stable after extubation.

CART technique (**C**lassification **A**nd **R**egression **T**ree) was used to create an algorithm with a combination of the tests, which qualified as an optimized predictor of the EMG. Briefly, CART divides the entire sample step by step into smaller binary subgroups. In each step, the sample is divided into two subgroups by investigating all possible splits and using the split with the best separation with respect to the dependent variable [20]. Both subgroups are independently analysed in the same way until either no significant split can be performed or the sample becomes too small. The dependent variable was the TOFR of the EMG-measurement.

To allow the comparison of the model based on function tests with the metric TOFR-values of EMG or AMG we dichotomised the TOFR in a way clinical decisions are typically made. As cut-off we defined a neuromuscular function of an EMG-measured TOFR = 0.9 and the formerly accepted level of TOFR = 0.7. The tactile TOFR was dichotomised in “fading palpable” or “fading not palpable”. The overall performance of the models for the different cut-off levels was calculated in form of receiver-operated characteristics (ROC). The areas under the curves (AUC) of the ROC curves were used as a measure for the discriminative power of the models.

For validation of the developed tools (uncalibrated AMG, tactile evaluation following nerve stimulation and algorithm of muscle function tests), sensitivity and specificity with exact binomial confidence intervals were calculated. Sample size was calculated based on the assumptions that the algorithm most probably will not be able to predict TOFR ≥0.9, but a TOFR ≥0.7 is a level of recovery of the neuromuscular function possibly sufficient to avoid major complications. Therefore, we primarily focused on a high sensitivity (>90%) with an accuracy of the estimate <10%, resulting in a necessary sample of at least 36 patients with TOFR <0.7 and at least 36 patients with TOFR ≥0.7. In the development cohort, 49% of patients had a TOFR ≥0.7 and 51% had a TOFR <0.7, 95%-confidence intervals reached from 41% to 59%. Therefore, we decided to include 100 patients for the validation assuming to result in at least 40 patients with TOFR <0.7 as well as at least 40 patients with TOFR ≥0.7.

The risk to overlook patients with a residual neuromuscular block with the three tools depends on the sensitivity (sens) and specificity (spec) of the tools and the prevalence (p(ε)) at the respective TOFR level *ε*. Since the validation part of the study was not designed to evaluate the prevalence of postoperative residual neuromuscular block, the risk (P) to overlook a residual block can be calculated dependent on the unknown prevalence only:

$$ P\left({TOF}_{EMG}<\varepsilon \left|{TOFR}_{tool}>\right.\varepsilon \right)=\frac{p\left(\varepsilon \right)\times \left(1- sens\right)}{p\left(\varepsilon \right)\times \left(1- sens\right)+\left(1-p\left(\varepsilon \right)\right)\times spec} $$

Statistics were performed using STATA (StataCorpLP, Texas, USA). Values are presented as means and 95% confidence interval.

## Results

### Development

For the algorithm development, 200 patients were enrolled (age: 41 ± 14 years; weight: 79 ± 23 kg; body mass index BMI: 25 ± 3 kg/m^2^). In 26 patients the EMG signal showed significant jerky leaps during emergence from anaesthesia (see e.g. Fig. 2), 9 patients had intraoral operations that did not allow swallowing water and the spatula pressure test. In 165 patients the EMG signal quality remained stable and the complete set of tests could be performed. The values of the two neuromuscular monitoring techniques and times are given in Table 2. Following assessment, 114 of the 165 patients required reversal of the neuromuscular block with neostigmine. After 30 min, no patient had any clinical sign of neuromuscular weakness, i.e. all clinical tests could be performed without any limitation.

**Table 2:** The values of the two neuromuscular monitoring techniques (EMG and AMG) during the algorithm development and validation at the different time points during the study. Values are given as mean ± SD (ranges)

	Algorithm development (*n* = 165)	Algorithm validation(*n* = 100)
T1/T0 after calibration(EMG)	0.96 ± 0.02(0.90–1.01)	0.96 ± 0.02(0.91–1.00)
T1/T0 at extubation(EMG)	0.64 ± 0.24(0.10–1.08)	0.62 ± 0.20(0.11–1.02)
TOFR at extubation(EMG)	0.57 ± 0.33(0.00^a^ – 1.03)	0.47 ± 0.25(0.00^a^ – 1.00)
T1/T0 at assessment(EMG)	0.68 ± 0.22(0.12–1.11)	0.66 ± 0.19(0.23–1.20)
TOFR at assessment(EMG)	0.61 ± 0.31(0.00^a^ – 1.15)	0.53 ± 0.25(0.00^a^ – 0.99)
TOFR at assessment(AMG)	0.63 ± 0.32(0.00^a^ – 1.20)	0.57 ± 0.25(0.00^a^ – 1.00)
TOFR after 30 min PACU(AMG)	0.96 ± 0.09^b^(0.66–1.26)	0.97 ± 0.09(0.92–1.02)

*EMG* electromyography, *AMG* acceleromyography, *PACU* post anaesthesia care unit

^a^T2/T0 > 0, i.e. reappearance of the second twitch response; ^b^ (*n* = 133)

**Fig. 3:**
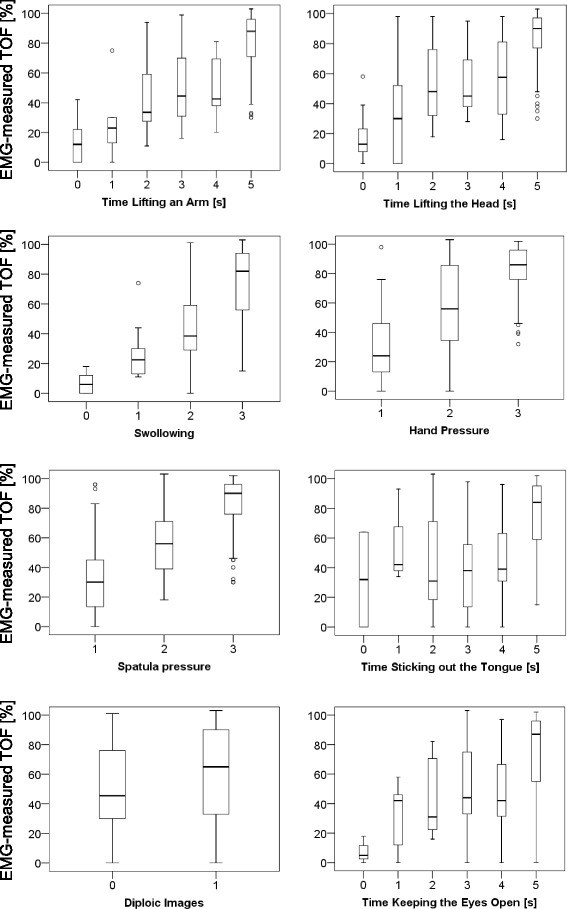
Specification of eight clinical tests in relation to the Train-of-Four Ratio (TOFR) as measured by electromyography

**Fig. 4:**
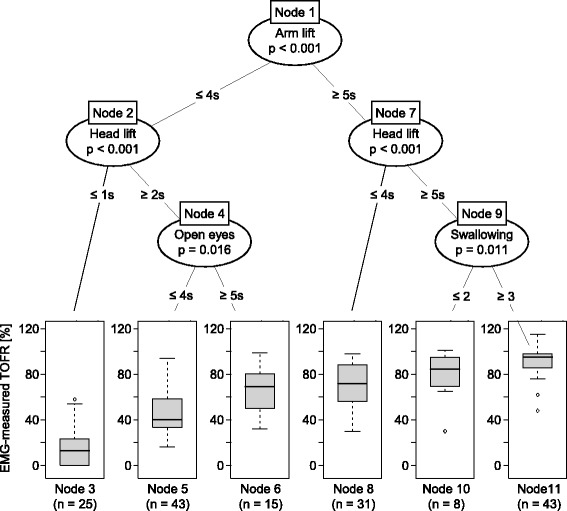
Regression analysis with classification and regression tree (CART). The upper section of the figure depicts how CART revealed six nodes of test scores that significantly divide the collective regarding the TOFR measured by calibrated electromyography (EMG). The lower section of the figure shows boxplots of EMG measured TOFR in patients allocated to the respective nodes. The test combinations of node 11 (arm lift ≥5 s, head lift ≥5 s, and swallowing without any hindrance) was able to discriminate between patients with TOFR <0.7 and TOFR ≥0.7

Patients with better muscle function tests had a tendency towards higher TOFRs. No single test was an acceptable predictor of the EMG-measured TOFR (Fig. 3). CART analysis showed that EMG-values could be predicted with three tests only (Fig. 4). In the first step, the variable “arm lift” divided the set of patients at a cut-off point of 5 s. Both subsets were further split by the variable “head lift”. Patients, who could not lift their arm for 5 s and their head for at least 2 s (node 3), had in 22 of 25 cases (88%) an EMG-measured TOFR <0.3. Patients who were able to lift the arm for 5 s, to lift the head for 5 s, and to swallow 20 ml of water without any restriction (node 11) had in 41 of 43 cases (95%) an EMG-measured TOFR >0.7 and in 30 cases (74%) an EMG-measured TOFR >0.9. Finally, CART resulted in six decision pathways (Fig. 4: node 3, node 5, node 6, node 8, node 10, and node 11).

**Fig. 5:**
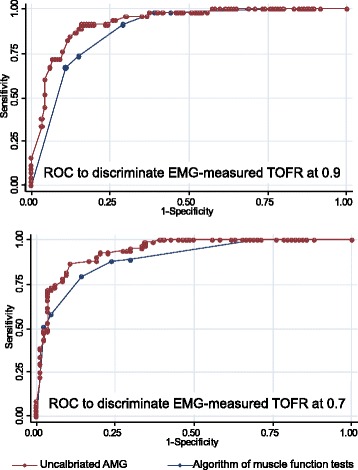
Receiver operated characteristic (ROC) curves to discriminate electromyography (EMG) measured train-of-four ratio (TOFR) with uncalibrated AMG and algorithm of muscle function tests. The area under the curves (AUC) of uncalibrated acceleromyography (AMG) and the algorithm of muscle function tests did not differ significantly for TOFR <0.7 (*p* = 0.094) as well as TOFR <0.9 (*p* = 0.136)

For cut-off points TOFR <0.9 and TOFR <0.7 ROC-curves were calculated and the corresponding values of the algorithm of muscle function tests were compared with AMG (Fig. 5). There was no significant difference between the AMG-measurement and the algorithm of muscle function tests regarding the AUC of the ROC curves.

### Validation

In order to validate the three tools to identify TOFR <0.9 and TOFR <0.7, a separate set of 118 patients were enrolled, of which 100 patients were analysed (age: 39 ± 12 years; weight: 76 ± 14 kg; body mass index (BMI) 25 ± 3 kg/m^2^). Two patients had to be excluded due to unexpected intraoral surgery and 16 because of loss of EMG signal quality during emergence from anaesthesia. The neuromuscular block at the time of assessment is given in Table 2. Following clinical assessment, 62 of the 100 patients required reversal with neostigmine. After 30 min in the PACU, no patient had clinical signs of neuromuscular weakness. Prediction coefficients reveal an advantage of the uncalibrated AMG compared to the algorithm of muscle function tests in terms of specificity to identify the EMG-measured TOFR <0.7 (89.7% vs. 34,5%) and TOFR <0.9 (100% vs. 42.9%). While the latter has a higher sensitivity in comparison to the uncalibrated AMG at TOFR <0.7 (100% vs. 94.4%) (Table 3).

**Table 3:** Validation of the muscle function algorithm (head lift, arm lift, swallowing 20 ml water, eye opening), tactile fading after peripheral nerve stimulation (PNS), and uncalibrated acceleromyography (AMG) to identify patients with TOF < 0.9 or TOF < 0.7 in the post anaesthesia care unit. Results (with 95% confidence intervals) from a second prospective cohort of 100 patients

		Algorithm of muscle function tests	Fading following PNS	Uncalibrated AMG
TOF < 0.9	Sensitivity	92.5% [85.1; 96.9]	33.7% [24.2; 44.3]	93.6% [86.5; 97.6]
	Specificity	42.9% [9.9; 81.6]	0.0% [0.0; 41.0]	100.0% [59.0; 100]
TOF < 0.7	Sensitivity	100% [94.9; 100]	18.8% [10.4; 30.1]	94.4% [86.2; 98.4]
	Specificity	34.5% [17.9; 54.3]	16.7% [5.6; 34.7]	89.7% [72.6; 97.8]

**Fig. 6:**
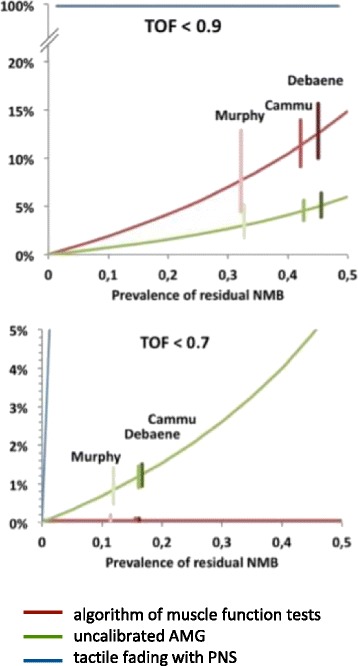
Risk to overlook patients with residual neuromuscular blockade with a TOFR <0.9 and TOFR <0.7 when assessed with either uncalibrated acceleromyography (AMG), the algorithm of clinical muscle function tests, or tactile fading following peripheral nerve stimulation (PNS). The lines represent the mean risk to overlook residual neuromuscular blockade as a function of its prevalence. Exemplarily, the risks (bars are the 95% confidence intervals) are marked based on the prevalence found by Debaene et al. [25] Cammu et al. [15] and Murphy et al. [18]

Figure 6 shows the calculated risk to ignore a residual block of TOFR <0.7 and TOFR <0.9 with a PNS, an uncalibrated AMG and the algorithm of muscle function tests. TOFR >0.9 is always ignored with PNS related techniques (risk = 100%) due to the specificity = 0 (Table 3) of the tool at this TOFR. Following an uncalibrated AMG measured TOFR >0.9, a patient has the lowest risk to actually have a TOFR <0.9. The sensitivity of the muscle function algorithm for TOFR <0.7 (=100%) reduces the risk to 0 for a patient, who successfully passes head lift, arm lift and swallowing, to be at TOFR <0.7.

## Discussion

Residual neuromuscular block in awake patients can be validly identified with an uncalibrated AMG. For the first time we could demonstrate that the combination of four muscle function tests, including duration of arm lift, duration of head lift, duration to keep the eyes open, and ability to swallow 20 ml of water, has a discriminative power comparable to the TOFR of an uncalibrated AMG. Based on three of these tests, an algorithm was developed, which is able to identify patients with a TOFR <0.7 and, with restrictions, a TOFR <0.9.

There is no doubt that calibrated, quantitative neuromuscular monitoring is the gold standard to measure neuromuscular function ***during*** anaesthesia and before extubation [21]. Intuitively, one may also prefer common quantitative neuromuscular monitoring in the PACU. In awakening or awake patients after anaesthesia, however, there is no evidence about its quality irrespectively its complex applicability [11, 13]. Nevertheless, the risk to overlook residual or reoccurrence of neuromuscular block ***after*** anaesthesia, i.e. in the PACU, necessitates a valid tool for differential diagnostic reasons [22, 23]. This is undergirded by the fact that even with the use of sugammadex without neuromuscular monitoring TOFR in the PACU still remains less than 0.9 in almost 9.4% [24].

The number of and the reasons for excluded patients in this study demonstrate the complexity of any neuromuscular monitoring technique in awake patients, even when continued after anaesthesia. All mandatory measures were applied to guarantee stable measurements of EMG in order to serve as reference method [19]. In addition, we performed the measurements during a 30 min extended stay in the operating room in order to reduce transport related failures. Nevertheless, we were not able to continue EMG much less to reliably start AMG monitoring in 26 patients (13.0%). In this cohort of alert, orientated and cooperative patients, the clinical assessment could not be completed in 4.5% of the patients only. The most difficult test to be carried out was the swallowing test.

In the patients, in whom the technical problems could be controlled, the AMG proved to reproduce the calibrated EMG in the best way. This is even more remarkable, since we mimicked the clinical conditions of a PACU tool, which cannot be calibrated before relaxation as a matter of principle. AMG, however, overestimated the TOFR measured by EMG. This well-known problem is responsible for the risk to overlook a TOFR <0.9 as well as a TOFR <0.7 with AMG (Fig. 6) [25]. Based on the prevalence reported by Debaene et al., Cammu et al., or Murphy et al. an average of 3% – 5% patients must be expected to have a TOFR <0.9, when AMG indicates a TOFR ≥0.9, and, more important, up to 1.6% would be at risk to have a TOFR <0.7, when AMG indicates a TOFR ≥0.7 (Fig. 6) [15, 18, 26]. Accordingly, we recommend increasing the requested level of TOFR recovery when using AMG in awake patients analogously to anaesthetised patients [27, 28].

Tactile neuromuscular monitoring cannot reveal a residual neuromuscular block with a TOFR between 0.4 and 0.9 in anaesthetised patients [29]. In our study, this missing discriminative power of tactile evaluation was confirmed in awake patients. As expected, tactile fading in the TOF-stimulation pattern is neither able to identify a TOFR <0.9 nor a TOFR <0.7 in awake patients (Fig. 6).

Studies investigating residual paralysis found a lack of accordance between individual muscle function tests and uncalibrated AMG monitoring [14, 15]. We confirmed these findings demonstrating that single tests reproduced the well-known varying sensitivity of muscles at arm, neck, pharynx, and eyes (Fig. 3) [30–33]. Therefore, we used CART to create a clinically practicable algorithm based on the muscle function tests. CART splits the entire sample into two subgroups out of all possible scores searching for the best separation [20]. As the resulting subgroups are analysed in the same way independently until either no significant split could be performed or the sample became too small, a hierarchical model is generated. The result of this modelling has the great advantage of easy applicability without any calculations or technical measurements, simply by logically combing the assessments. Therefore, minor lack of discriminative power may be acceptable, because the clinical practicability effectively increases.

The upper airway function played a key role, when the level of acceptable TOFR recovery was defined to be 0.9 based on a number of sophisticated studies in volunteers [32, 34–36]. After a successfully performed 5 s arm and a 5 s head lift (node 1, node 7), expectedly, the simplistic test to swallow 20 ml water was not able to discriminate between TOFR <0.9 and TOFR >0.9 alone. Nevertheless, the swallowing test contributed to the algorithm exactly at this TOFR (node 9, Fig. 4).

The high sensitivity (100%) of the algorithm to identify patients with severe residual paralysis (TOFR <0.7) may protect affected patients in the PACU to be overlooked, independent how many patients actually have a TOFR <0.7 (Fig. 6). Since data on respiratory failure support the notion that TOFR <0.7 indicates patients, who are at high and acute risk, the algorithm seems to be an improvement in terms of safety, identifying severe residual paralysis [2].

The high sensitivity of the algorithm, however, also implies the risk to overestimate residual neuromuscular block and therefore provoke overtreatment with its immanent side effects. High dose cholinesterase inhibitors like neostigmine applicated at minimal levels of neuromuscular block, e.g. may induce nausea and vomiting, increase airway secretion, and may paradoxically impair the upper airway muscle function [37, 38].

Residual block with TOFR <0.3 should be recognised before extubation even without any neuromuscular monitoring. But neither pharmacological reversal with neostigmine nor with sugammadex based on non-systematic clinical signs of muscle weakness are able to avoid PORC [39]. It was not our aim to provoke extubation at such deep levels. The members of the study staff, however, routinely apply quantitative neuromuscular monitoring when paralyzing their patients and, therefore, were less experienced to work without it. Nevertheless, no patient was harmed by one of the typical complications of a residual block. Although there was a potential risk of pulmonary aspiration using the swallowing test (20 ml of water), we observed no makro aspiration. Mikro aspiration could not be excluded clinically. This might be due to the short assessment period before reversal with 40 μg/kg neostigmine, the thorough care taking by the study staff expecting patients with a residual block, but also the patients’ preoperative information that such a scenario might happen.

There are limitations to the present investigation. First, the algorithm was developed in a well-defined group of patients (ASA 1, 2) without organ dysfunction. Second, the relatively young study population (18–65 years) was scheduled just for elective, low risk, surgical procedures. Third, just short-acting anesthetic medication (desflurane, remifentanil) was used, enabling sufficient alertness and cooperation after extubation for the clinical assessment. Fourth, swallowing of 20 ml water as a part of the algorithm might provoke aspiration in patients with residual paralysis.

## Conclusion

We developed and verified tools for the PACU to identify patients with residual neuromuscular block. AMG, even when used uncalibrated in awakening patients, proved to identify a residual neuromuscular block. An algorithm based on muscle function tests is also able to indicate residual neuromuscular blocks with high sensitivity for a TOFR <0.7. This tool might reduce the risk to overlook severe residual neuromuscular block in the PACU of institutions, in which only qualitative neuromuscular monitoring with PNS is used.

Further clinical studies are necessary to test this muscle function algorithm in other populations (ASA 3–4, age > 65 years) and varying clinical settings.

## Abbreviations

AMG: Acceleromyography
ASA: American society of anaesthesiologists
AUC: Area under the curves
CART: Classification and regression tree
EMG: Electromyography
PACU: Postoperative care unit
PNS: Peripheral nerve stimulators
PORC: Postoperative residual curarization
ROC: Receiver-operated characteristics
Sens: Sensitivity
Spec: Specifity
T1/T0: First stimulation related to the baseline value
TOF: Train-of-four
TOFR: Train-of-four ratio

## References

[1] Baillard C, Clec’h C, Catineau J, Salhi F, Gehan G, Cupa M, Samama CM (2005). Postoperative residual neuromuscular block: a survey of management. Br J Anaesth.

[2] Murphy GS, Szokol JW, Marymont JH, Greenberg SB, Avram MJ, Vender JS, Nisman M (2008). Intraoperative acceleromyographic monitoring reduces the risk of residual neuromuscular blockade and adverse respiratory events in the postanesthesia care unit. Anesthesiology.

[3] Fuchs-Buder T, Fink H, Hofmockel R, Geldner G, Ulm K, Blobner M (2008). Einsatz des neuromuskularen Monitorings in Deutschland. Anaesthesist.

[4] Duvaldestin P, Cunin P, Plaud B, Maison P (2008). French survey of neuromuscular relaxant use in anaesthetic practice in adults. Ann Fr Anesth Reanim.

[5] Grayling M, Sweeney BP (2007). Recovery from neuromuscular blockade: a survey of practice. Anaesthesia.

[6] Sorgenfrei IF, Viby-Mogensen J, Swiatek FA (2005). Does evidence lead to a change in clinical practice? Danish anaesthetists’ and nurse anesthetists’ clinical practice and knowledge of postoperative residual curarization. Ugeskr Laeger.

[7] American Society of Anesthesiologists. Standards for basic anesthetic monitoring (approved by house of delegates on October 21, 1986, and last amended on October 15, 2003). ASA Standards, Guidelines and Statements. 2003.

[8] Deutsche Gesellschaft für Anästhesiologie und Intensivmedizin e.V. und Berufsverband Deutscher Anästhesisten e.V: Mindestanforderungen an den anästhesio­ logischen Arbeitsplatz. *Anästh Intensivmed.* 2013;54:39–42.

[9] [Consensus conference: Indications for curarization in anesthesia. Saint-Mande, 8 July 1999. Proceedings]. *Ann Fr Anesth Reanim.* 2000;19 Suppl 2:344s–472s. 11203410

[10] Eriksson LI (2003). Evidence-based practice and neuromuscular monitoring: it’s time for routine quantitative assessment. Anesthesiology.

[11] Kempen PM (2004). Obligate acceleromyography and pharmacologic reversal of all neuromuscular blocking agents: really, and where is the clinical outcome?. Anesthesiology.

[12] Naguib M, Kopman AF, Ensor JE (2007). Neuromuscular monitoring and postoperative residual curarisation: a meta-analysis. Br J Anaesth.

[13] Baillard C, Bourdiau S, Le Toumelin P, Ait Kaci F, Riou B, Cupa M, Samama CM (2004). Assessing residual neuromuscular blockade using acceleromyography can be deceptive in postoperative awake patients. Anesth Analg.

[14] Debaene B, Plaud B, Dilly MP, Donati F (2003). Residual paralysis in the PACU after a single intubating dose of nondepolarizing muscle relaxant with an intermediate duration of action. Anesthesiology.

[15] Cammu G, De Witte J, De Veylder J, Byttebier G, Vandeput D, Foubert L, Vandenbroucke G, Deloof T (2006). Postoperative residual paralysis in outpatients versus inpatients. Anesth Analg.

[16] Murphy GS, Szokol JW, Marymont JH, Greenberg SB, Avram MJ, Vender JS (2008). Residual neuromuscular blockade and critical respiratory events in the postanesthesia care unit. Anesth Analg.

[17] Hayes AH, Mirakhur RK, Breslin DS, Reid JE, McCourt KC (2001). Postoperative residual block after intermediate-acting neuromuscular blocking drugs. Anaesthesia.

[18] Murphy GS, Szokol JW, Avram MJ, Greenberg SB, Shear T, Vender JS, Gray J, Landry E (2013). Postoperative residual neuromuscular blockade is associated with impaired clinical recovery. Anesth Analg.

[19] Fuchs-Buder T, Claudius C, Skovgaard LT, Eriksson LI, Mirakhur RK, Viby-Mogensen J (2007). Good clinical research practice in pharmacodynamic studies of neuromuscular blocking agents II: the Stockholm revision. Acta Anaesthesiol Scand.

[20] Hothorn T, Hornik K, Zeileis A (2006). Unbiased Recursive Partitioning: A Conditional Inference Framework. J Comput Graph Stat.

[21] Claudius C, Viby-Mogensen J (2008). Acceleromyography for use in scientific and clinical practice: a systematic review of the evidence. Anesthesiology.

[22] Le Corre F, Nejmeddine S, Fatahine C, Tayar C, Marty J, Plaud B (2011). Recurarization after sugammadex reversal in an obese patient. Can J Anaesth.

[23] Unterbuchner C, Fink H, Berthele A, Blobner M (2014). Case scenario: residual curarization in diabetic polyneuropathy. Anesthesiology.

[24] Kotake Y, Ochiai R, Suzuki T, Ogawa S, Takagi S, Ozaki M, Nakatsuka I, Takeda J (2013). Reversal with sugammadex in the absence of monitoring did not preclude residual neuromuscular block. Anesth Analg.

[25] Liang SS, Stewart PA, Phillips S (2013). An ipsilateral comparison of acceleromyography and electromyography during recovery from nondepolarizing neuromuscular block under general anesthesia in humans. Anesth Analg.

[26] Debaene B, Plaud B, Dilly M-P, Donati F (2003). Residual paralysis in the PACU after a single intubating dose of nondepolarizing muscle relaxant with an intermediate duration of action. Anesthesiology.

[27] Eikermann M, Groeben H, Husing J, Peters J (2003). Accelerometry of adductor pollicis muscle predicts recovery of respiratory function from neuromuscular blockade. Anesthesiology.

[28] Capron F, Alla F, Hottier C, Meistelman C, Fuchs-Buder T (2004). Can acceleromyography detect low levels of residual paralysis? A probability approach to detect a mechanomyographic train-of-four ratio of 0.9. Anesthesiology.

[29] Viby-Mogensen J, Jensen NH, Engbaek J, Ording H, Skovgaard LT, Chraemmer-Jørgensen B (1985). Tactile and visual evaluation of the response to train-of-four nerve stimulation. Anesthesiology.

[30] Ali HH, Utting JE, Gray TC (1971). Quantitative assessment of residual antidepolarizing block. II. Br J Anaesth.

[31] Ali HH, Utting JE, Gray TC (1971). Quantitative assessment of residual antidepolarizing block. I. Br J Anaesth.

[32] Eikermann M, Vogt FM, Herbstreit F, Vahid-Dastgerdi M, Zenge MO, Ochterbeck C, de Greiff A, Peters J (2007). The predisposition to inspiratory upper airway collapse during partial neuromuscular blockade. Am J Respir Crit Care Med.

[33] Hemmerling TM, Schmidt J, Hanusa C, Wolf T, Schmitt H (2000). Simultaneous determination of neuromuscular block at the larynx, diaphragm, adductor pollicis, orbicularis oculi and corrugator supercilii muscles. Br J Anaesth.

[34] Sundman E, Witt H, Olsson R, Ekberg O, Kuylenstierna R, Eriksson LI (2000). The incidence and mechanisms of pharyngeal and upper esophageal dysfunction in partially paralyzed humans: pharyngeal videoradiography and simultaneous manometry after atracurium. Anesthesiology.

[35] Kopman AF, Yee PS, Neuman GG (1997). Relationship of the train-of-four fade ratio to clinical signs and symptoms of residual paralysis in awake volunteers. Anesthesiology.

[36] Eriksson LI, Sundman E, Olsson R, Nilsson L, Witt H, Ekberg O, Kuylenstierna R (1997). Functional assessment of the pharynx at rest and during swallowing in partially paralyzed humans: simultaneous videomanometry and mechanomyography of awake human volunteers. Anesthesiology.

[37] Tramer MR, Fuchs-Buder T (1999). Omitting antagonism of neuromuscular block: effect on postoperative nausea and vomiting and risk of residual paralysis. A systematic review. Br J Anaesth.

[38] Eikermann M, Zaremba S, Malhotra A, Jordan AS, Rosow C, Chamberlin NL (2008). Neostigmine but not sugammadex impairs upper airway dilator muscle activity and breathing. Br J Anaesth.

[39] Nemes R, Fülesdi B, Pongracz A, Asztalos L, Szabo-Maak Z, Lengyel S, Tassonyi E (2016). Impact of reversal strategies on the incidence of postoperative residual paralysis after rocuronium relaxation without neuromuscular monitoring. A partially randomised placebo controlled trial. Eur J Anaesthesiol.

